# Malignant cell receptor-ligand subtypes guide the prediction of prognosis and personalized immunotherapy of liver cancer

**DOI:** 10.18632/aging.205453

**Published:** 2024-01-18

**Authors:** Junzheng Wu, Chuncheng Wu, Xianhui Cai, Peipei Li, Jianjun Lin, Fuqiang Wang

**Affiliations:** 1 Xiamen Hospital of Traditional Chinese Medicine, Xiamen Hospital, Beijing University of Chinese Medicine, Xiamen, Fujian, China; 2 Xiamen Xianyue Hospital, Xiamen, Fujian, China

**Keywords:** hepatocellular carcinoma, scRNA-seq, immunotherapy, risk-score

## Abstract

Objective: Liver cancer is a prevalent disease with a dismal prognosis. The aim of the research is to identify subgroups based on malignant cell receptor ligand gene from single-cell RNA, which might lead to customized immunotherapy for patients with liver cancer.

Methods: Based on scRNA-seq data, we identified the receptor-ligand genes associated with prognosis and classify patients into molecular subtypes by univariate Cox regression and consensus clustering. LASSO regression was performed to construct a prognostic model, which was validated in TCGA and ICGC datasets. Immune infiltration and prediction of immunotherapy response were analyzed using ssGSEA, ESTIMATE, TIDE, and TRS score calculation. Finally, qPCR and Western blot validation of key genes and protein levels in cell lines.

Results: A risk model using 16-gene expression levels predicted liver cancer patients’ prognosis. The RiskScore associated significantly with tumor clinical characteristics and immunity, integrated with clinicopathological features for survival prediction. Differential expression of SRXN1 was verified in hepatocellular carcinoma and normal liver cells.

Conclusion: Our study utilizes single-cell analysis to investigate the communication between malignant cells and other cell types, identifying molecular subtypes based on malignant cell receptor ligand genes, offering new insights for the development of personalized immunotherapy and prognostic prediction models.

## INTRODUCTION

Hepatocellular carcinoma (HCC), ranking sixth highest in incidence and third in mortality rates, constitutes a significant global public health concern [1]. Its prognosis is often bleak, as the disease is frequently detected in its advanced stages, contributing to a five-year survival rate that is disappointingly low [2]. The complex nature of HCC, along with the heterogeneity amongst patients, presents substantial challenges to effective treatment strategies, with no universally effective therapy currently available [3]. Hence, the exploration and investigation of HCC carries profound significance for the health of populations worldwide.

A variety of therapeutic strategies are utilized in the treatment of HCC, including surgical interventions [1], radiofrequency ablation (RFA) or microwave ablation (MWA), systemic chemotherapy, Radiation therapy, and targeted therapies [4]. Recent advancements have also been made in immunotherapies, notably immune checkpoint inhibitors such as nivolumab and pembrolizumab [5]. Immune checkpoint inhibitors have been studied as a promising treatment option for liver cancer, but finding reliable biomarkers to predict response remains challenging [6]. The utilization of these medicines is currently limited to patients with advanced HCC, and they show variable responses with some patients achieving substantial durable responses, while many others do not respond or develop resistance after an initial response.

Currently, the malignant cell receptor ligand genes refer to those genes that encode proteins serving as ligands for receptors found on HCC cells. These ligands can bind to their corresponding receptors, leading to the activation of signaling pathways involved in the progression and survival of malignant cells [7]. Ligand-receptor interactions in liver cancer profoundly affect the immune microenvironment through various mechanisms in various studies. One previous study revealed that tumor cells continuously produce kynurenic acid (Kyn), an endogenous ligand for the aryl hydrocarbon receptor (AHR), via tryptophan-2,3-dioxygenase (TDO). Consequently, Kyn, acting through AHR, suppresses anti-tumor immune responses and promotes tumor cell survival and motility [8]. Furthermore, the transforming growth factor-beta (TGFβ) signaling pathway, known for its critical role in tumor development, influences processes like cell invasion and immune modulation [9]. Interestingly, the genetic characteristics of tumors are correlated with immune cell infiltration and the presence of neoantigens [10].

The objective of this study is to identify molecular subtypes of liver cancer based on malignant cell receptor ligand genes, and to develop a prognostic model for patients with liver cancer. To improve personalized immunotherapy, molecular subtypes based on malignant cell receptor ligand genes have been proposed as a potential approach.

## RESULTS

### Single-cell RNA clustering

**Figure 1:**
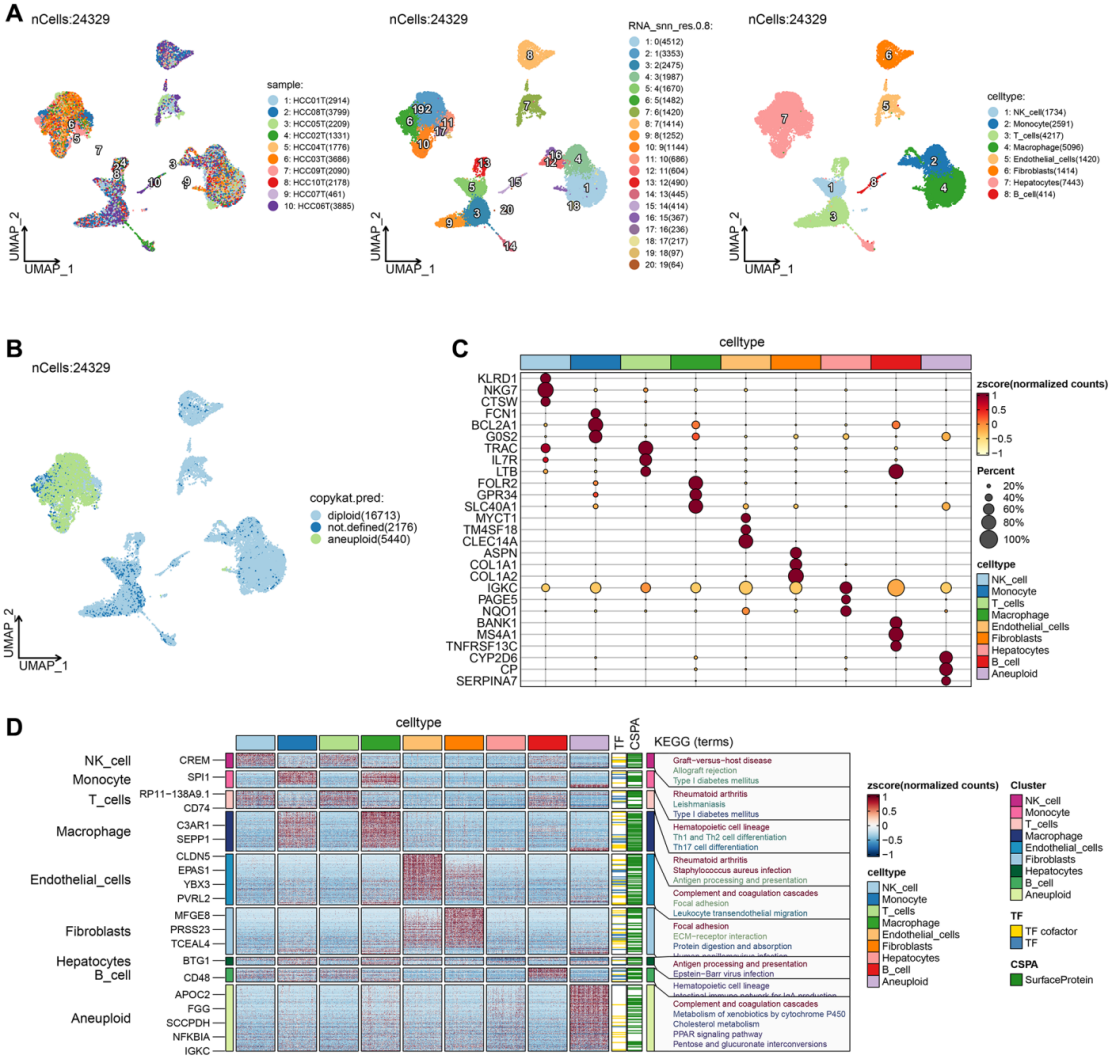
**Single-cell RNA clustering.** (**A**) UMAP plot of the distribution of 10 samples, UMAP plot of the distribution of 20 subpopulations, and UMAP plots of the subpopulations after cell annotation. (**B**) After CNV comment, the proportion and cell number of the subpopulation in each sample, and the UMAP map of malignant cells. (**C**) Dot plots of the expression of the first 5 marker genes of the subpopulations after CNV annotation. (**D**) The differential gene enrichment analysis of each subpopulation of cells.

After rigorous quality control, we retained 24,329 cells, comprising eight cell subpopulations (Figure 1A). The number of UMIs and mRNAs showed a significant correlation, while the number of UMI/mRNAs was not significantly correlated with the content of mitochondrial genes (Supplementary Figure 1A). Supplementary Figure 1B, 1C depict the violin plots before and after quality control, and Supplementary Figure 1D demonstrates significant elimination of batch effects between samples.

UMAP dimensionality reduction analysis was performed on 24,329 cells using RunUMAP function, revealing the presence of eight cell types: B cell, Endothelial cells, Hepatocytes, Macrophages, Monocytes, NK cell, Fibroblasts, and T cells. Aneuploid cells were included in cell cluster marker identification for ten cell types. We further analyzed the 10 samples based on the proportion of the ten subpopulations and identified the location of aneuploid malignant cells on the UMAP (Figure 1B). The top-five marker genes with the most outstanding contribution in each subgroup are shown in Figure 1C, and the results of marker genes are provided in Supplementary Table 1. The differential gene enrichment analysis of each subpopulation of cells is shown in Figure 1D, and the aneuploid cells differential genes were mainly enriched in the complement and coagulation cascades, cholesterol metabolism, PPAR signaling pathway, and pentose and glucuronate interconversion.

### Cellchat analysis and construction of cellular communication network

After rigorous quality control, we retained 49,701 cells, comprising 22,920 cancer cells and 26,781 normal liver tissue cells. Uniform manifold approximation and projection (UMI) and the number of mRNAs showed significant correlation, while there was no significant correlation between number of UMI/mRNAs and the content of mitochondrial genes, as seen in Supplementary Figure 1A. The violin plot before and after quality control can be seen in Supplementary Figure 1B–1D demonstrates significant elimination of batch effects between samples.

**Figure 2:**
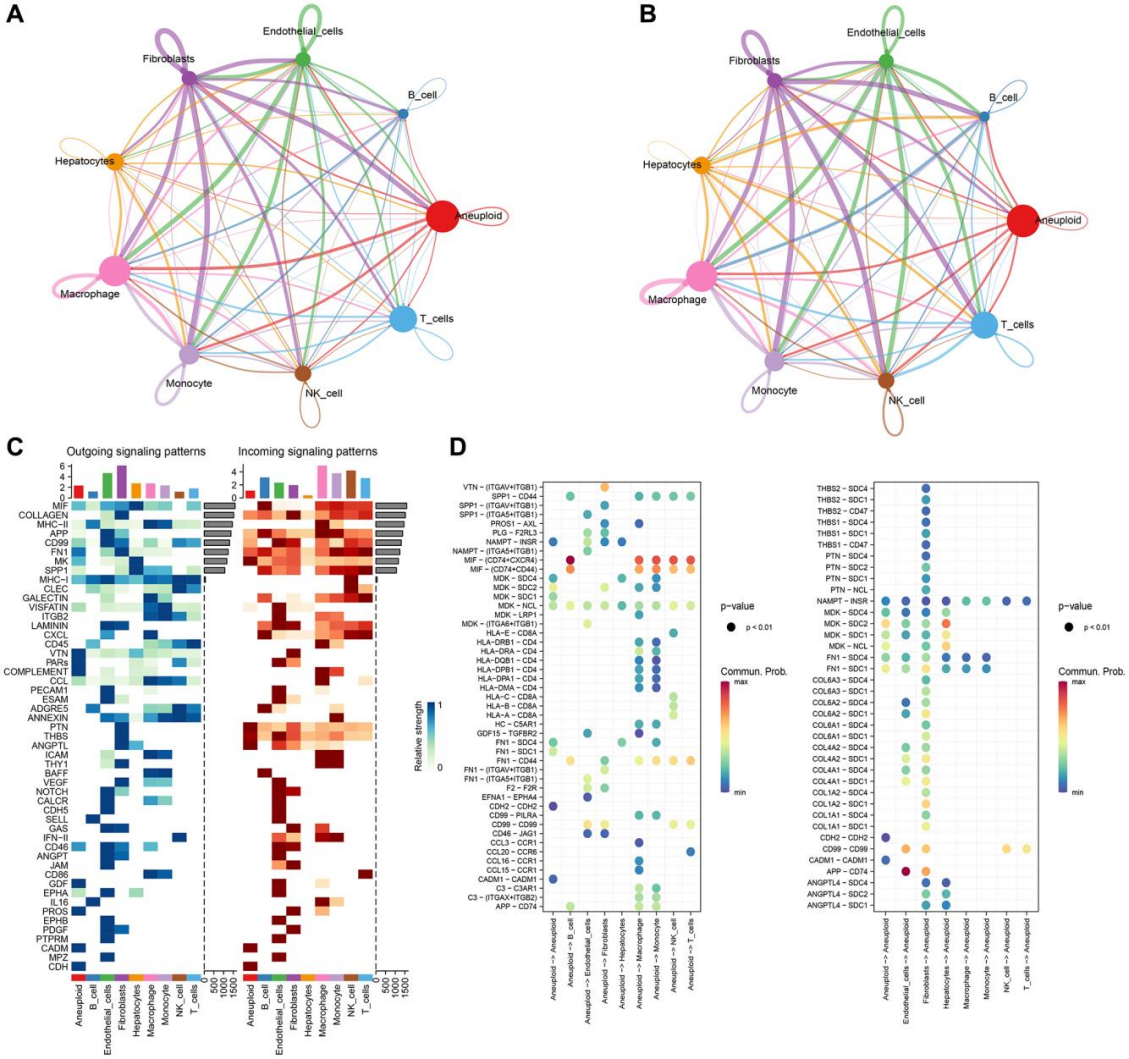
**CellChat analysis and construction of cellular communication network.** (**A**) Circle diagrams of that numb and strength of subpopulation interaction. (**B**) Number of communication interactions of each subgroup. (**C**) Communication and interaction strength of each subgroup. (**D**) Aneuploidy cell subpopulations and ligand receptor profiles among cell subpopulations.

We used copykat to identify malignant cells and analyzed the intersubpopulation communication network using the CellChat (version 1.5.0) package. Results showed that the communication between malignant cells and macrophages had the highest number and intensity (Figure 2A, 2B). Additionally, visual analysis revealed that the first six signaling pathways (MIF, MHC-II, APP, MHC-I) of subgroup communication had signal outputs of malignant cells, with the MIF signaling pathway having the strongest output and Monocyte being the main signal receiver cell (Figure 2C, 2D).

### Construction of molecular subtypes based on malignant cell receptor ligand genes

**Figure 3:**
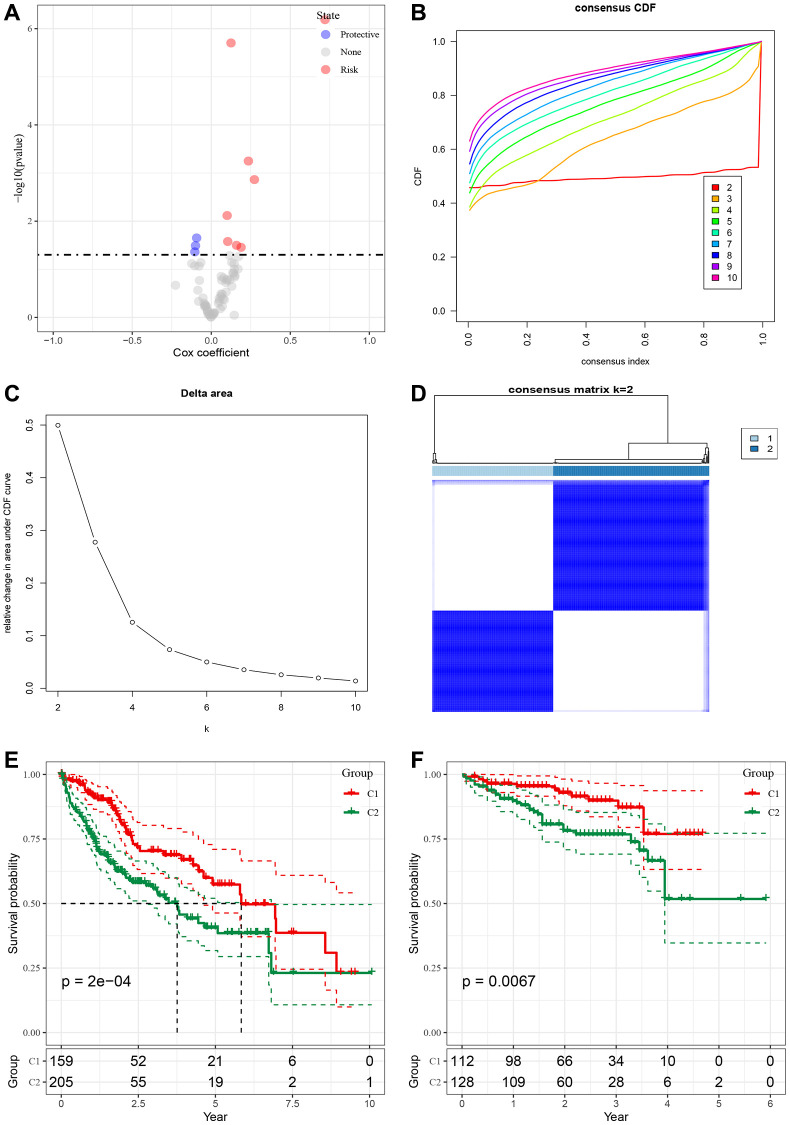
**Construction of molecular subtypes based on malignant cell receptor ligand genes.** (**A**) Single factor Cox analysis HR distribution map of malignant cell associated receptor gene set. (**B**) CDF curve of TCGA cohort sample. (**C**) CDF Delta area curve of TCGA cohort sample, the horizontal axis represents the number of clusters K, and the vertical axis represents the relative change of the area under the CDF curve. (**D**) Sample cluster heat map of consensus K = 2. (**E**) KM curve of prognosis relationship between two subtypes of TCGA. (**F**) KM curve of prognosis relationship between two subtypes of ICGC-JP.

To analyze the role of malignant cell-associated receptors in Bulk RNA-seq data, we screened 72 malignant cell-associated receptor genes using the results of the single-cell dataset CellChat. We found that 7 of the 72 related genes were associated with prognosis (*p* < 0.05), and protective gene 3 and risk gene 4 are shown in Figure 3A. We observed a relatively stable clustering result for Cluster 2 in the cumulative distribution function (CDF) Delta area curve (Figure 3B, 3C) and selected K = 2 to obtain two molecular subtypes (Figure 3D). Furthermore, analysis of the prognostic characteristics of these two molecular subtypes revealed a significant difference in prognosis (Figure 3E), with intersecting clust2 and clust1 subtypes having worse prognosis. The tcga. Subtype. CLI. Txt contains data for the TCGA dataset subtype. We performed the same clustering method on the independent ICGC-JP dataset and obtained similar results (Figure 3F). The icgc. Subtype. CLI table provides data on subtypes in the ICGC-JP dataset.

### Mutation characteristics of molecular subtypes

**Figure 4:**
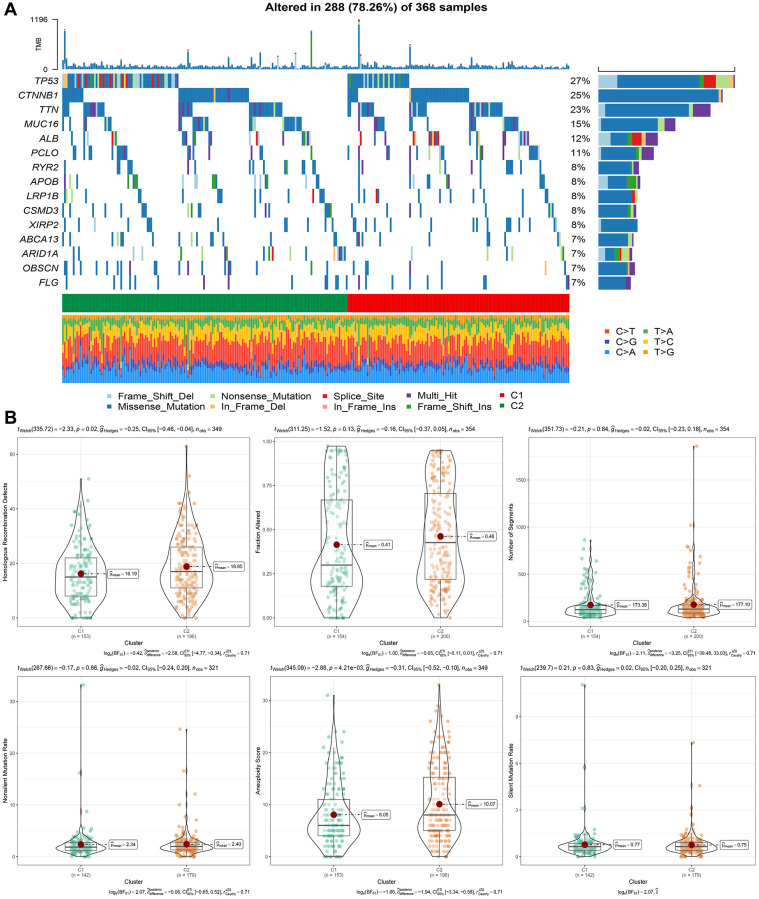
**Genomic alterations in molecular subtypes of the TCGA cohort.** (**A**) Somatic mutation analysis of different molecular subtypes in the TCGA cohort. (**B**) Comparison of “Homologous Recombination Defects”, “Fraction Altered”, “Number of Segments” “in different molecular subtypes of the TCGA cohort”. “Nonsilent Mutation Rate”, “Aneuploidy Score”, “difference in Silent Mutation Rate”.

We obtained SNV mutation data from TCGA using Mutect2, as shown in Figure 4A, and presented the top 15 genes with the most significant mutations in each subtype. Moreover, we surveyed “Homologous Recombination Defects”, “Fraction Altered”, “Number of Segments”, “Nonsilent Mutation Rate”, and “Aneuploidy Score,” as well as the distribution of the Silent Mutation Rate. We noticed that the Homologous Recombination Defects and Aneuploidy scores varied between subtypes, as demonstrated in Figure 4B (PMC5982584).

### Pathway analysis of molecular subtypes

**Figure 5:**
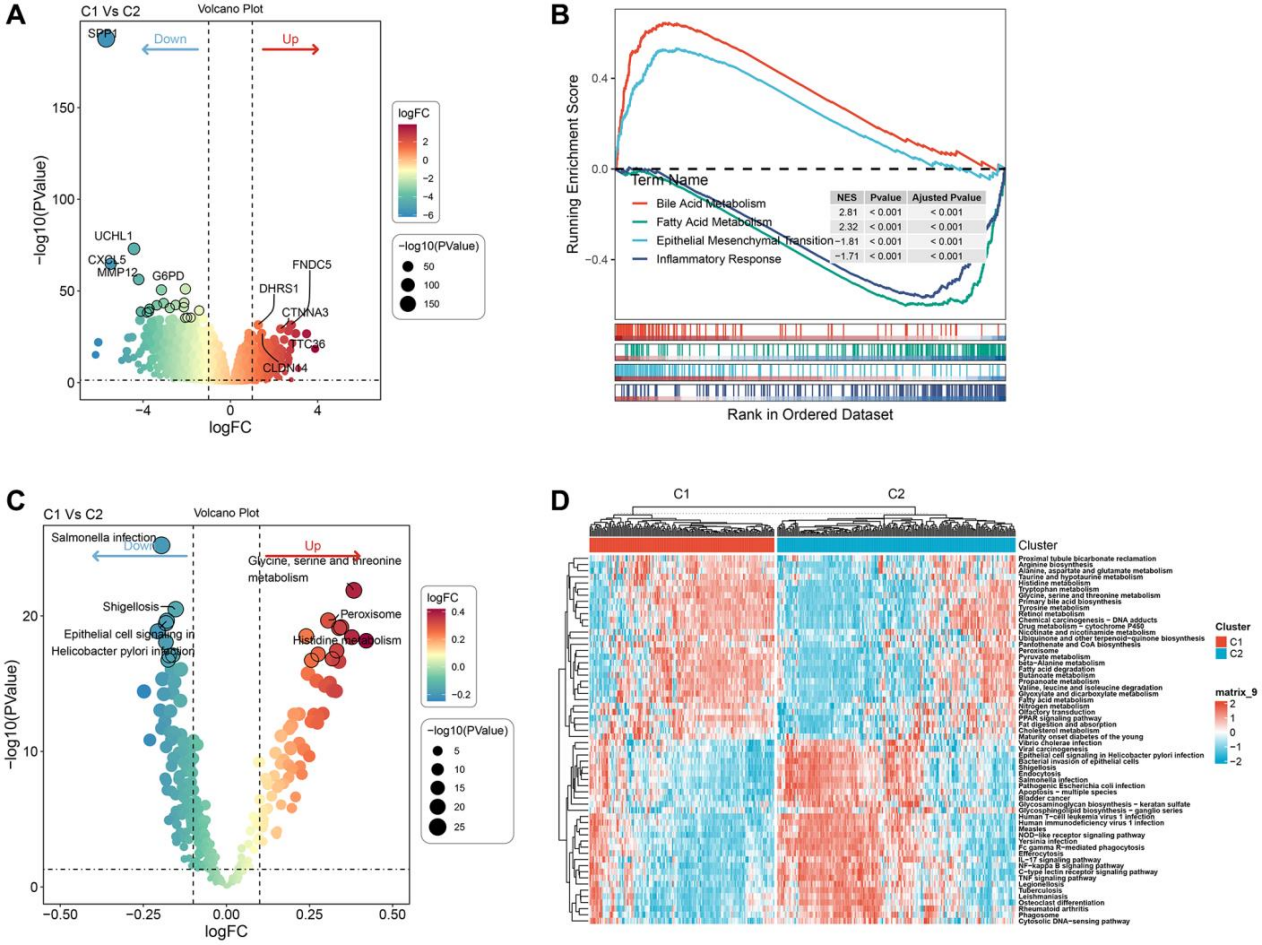
**Pathway analysis of molecular subtypes.** (**A**) Volcano map of differential analysis of genes between the two subtypes. (**B**) GSEA-GO analysis of differential genes between the two groups. (**C**) Bubble map of related pathways activated/inhibited in the comparison of the two subtypes. (**D**) Heat map of KEGG-related pathways with differences between the two subtypes.

We involved performing differential analysis for TCGA gene expression profiles grouped by cluster subtypes. This analysis revealed that when comparing clust1 to clust2, the expression of 188 genes increased while 1083 genes exhibited decreased expression (Figure 5A). Using the Hallmark gene sets in the Misgbd database as the background set, we performed a difference analysis GSEA (Figure 5B). The analysis results revealed that the Peroxisome and Histidine metabolism were activated in the clust1 subtype, whereas *Salmonella* infection, Shigellosis, and Epithelial cell signaling in *Helicobacter pylori* infection were inhibited, as demonstrated in Figure 5C. Our next step utilized GSVA pathway scoring on KEGG pathway enrichment obtained from the Misgbd database. Our analysis showed that 33 pathways were up-regulated and 23 were down-regulated, as demonstrated in Figure 5D.

### Immunological characteristics of molecular subtypes and differences in immunotherapy/chemotherapy

**Figure 6:**
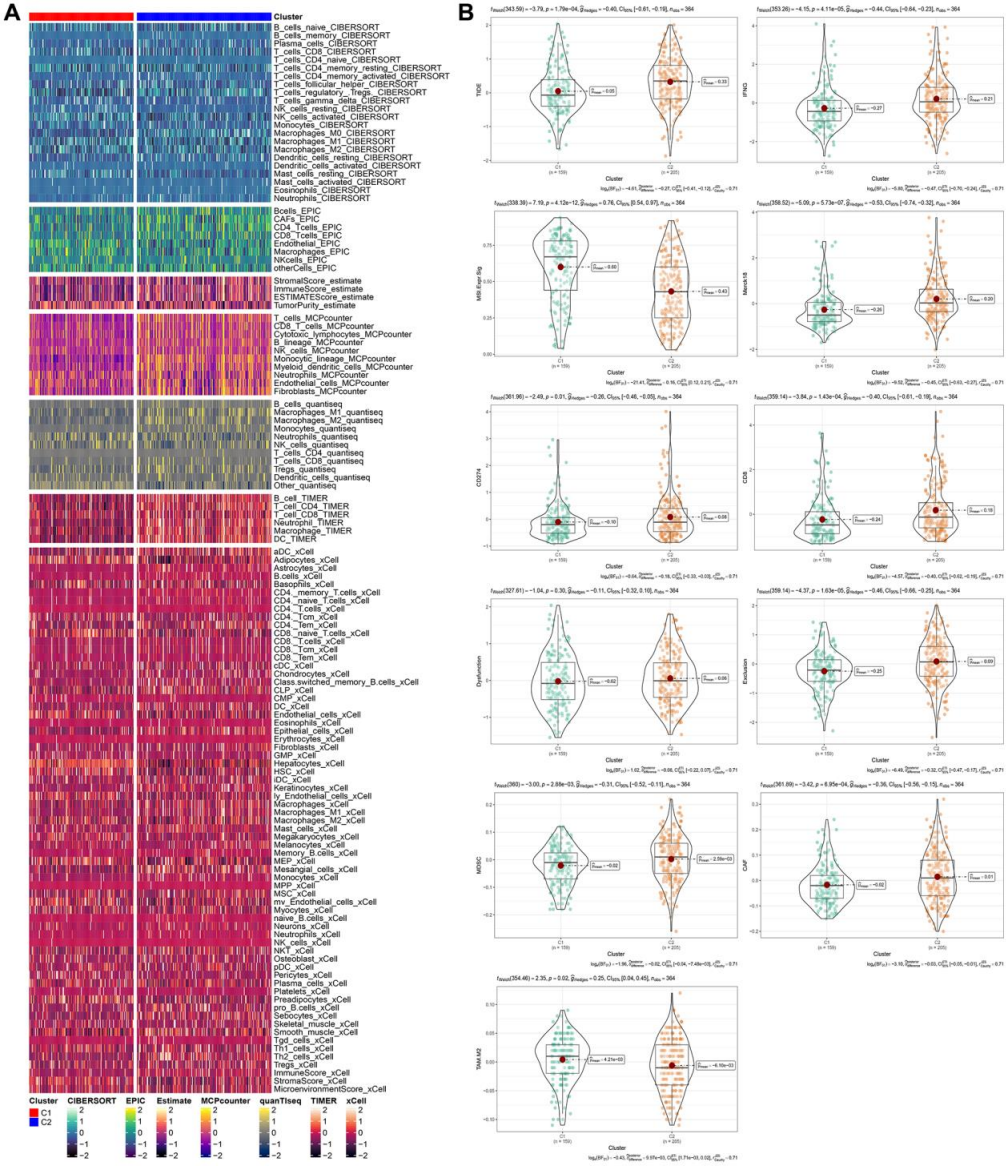
**Immunological characteristics of molecular subtypes and differences in immunotherapy/chemotherapy.** (**A**) The difference of 28 immune cell scores between different molecular subtypes in the TCGA cohort. (**B**) The difference of ESTIMATE immune infiltration between different molecular subtypes in the TCGA cohort. The immune checkpoint of differential expression between different subgroups in the TCGA cohort. Difference of TIDE analysis results between different groups in TCGA queue.

To determine the differences in immune microenvironment among molecular subtypes, we evaluated the infiltration level of immune cells using the expression levels of genes in immune cells in the TCGA cohort. We calculated the scores of immune cells using CIBERSORT, EPIC, Estlmate, MCPcounter, quanTlseg, TIMER, and xCell. Results indicated that the majority of immune cells had noteworthy differences between the two subtypes, with higher scores observed in clust2 (Figure 6A), which indicates that clust2 (with poor prognosis) exhibited a high immune score. We also looked at the expression levels of immune checkpoint genes in the two subtypes and discovered that there were significant variations between the two subtypes, with clust1 having a higher expression level than clust2 (Figure 6B). Patients with high TIDE prediction scores are unlikely to benefit from immunotherapy, indicating a higher probability of immune escape. A Wilcox Test on Figure 6B showed that the TIDE score was highest in the TCGA cohort’s clust2 subtype, suggesting that this subtype has an elevated likelihood of immune escape and is less likely to benefit from immunotherapy.

### Construction of risk model

**Figure 7:**
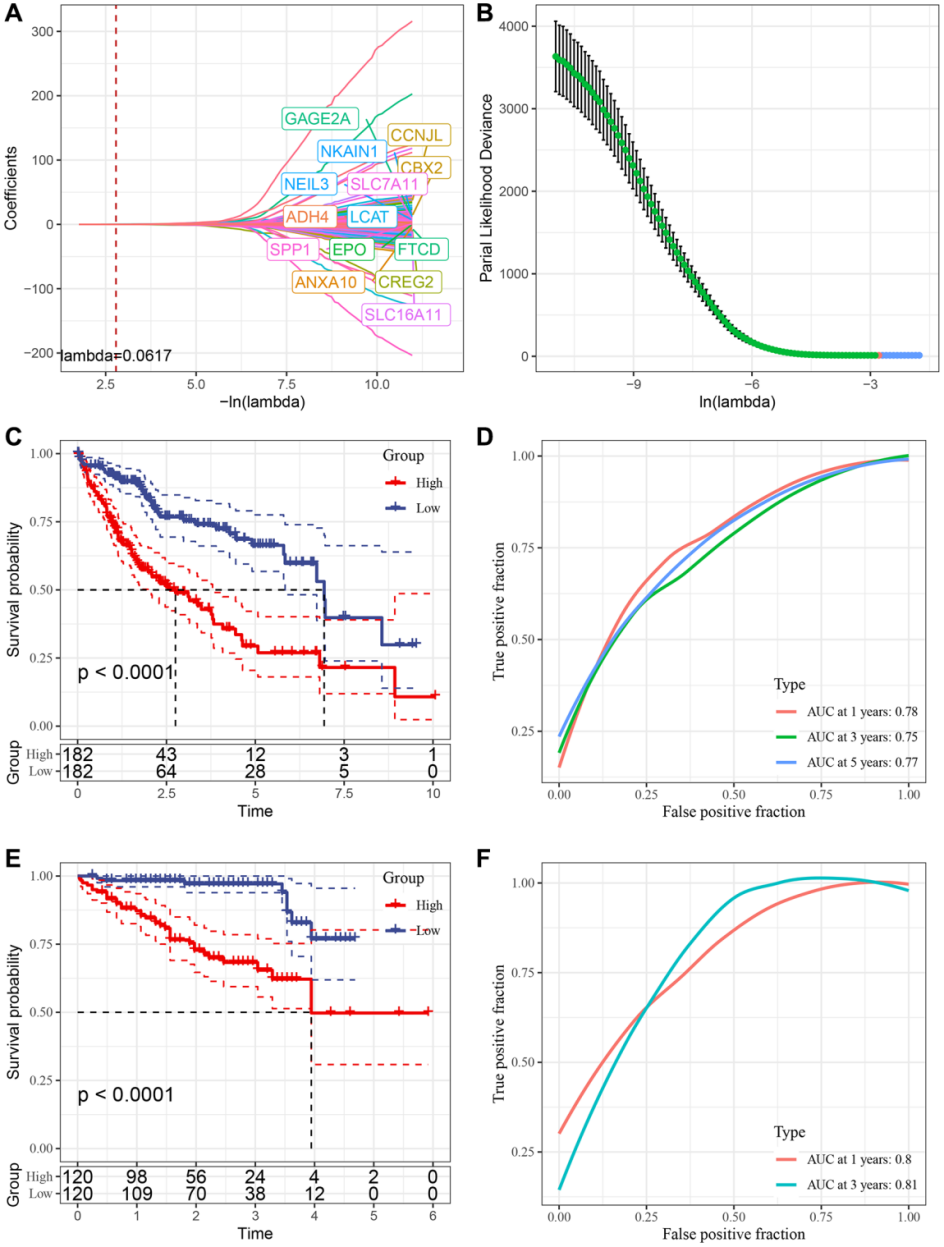
**Construction of risk model.** (**A**) Trajectory of each independent variable with lambda; (**B**) Confidence interval under lambda. (**C**) KM curve of high and low risk of risk model constructed by 16 genes in TCGA data set. (**D**) ROC curve of risk model constructed by 16 genes in TCGA data set. (**E**) KM curve of high and low risk of risk model built by 16 gene in ICGC data set. (**F**) ROC curve of risk models built by 16 gene in ICGC data set.

We conducted Cox regression analysis on the differentially expressed genes among the cluster subtypes and identified 285 prognostically relevant genes present in both the TCGA and ICGC datasets. Using Lasso regression, we compressed the 285 genes further in the TCGA dataset by reducing the number of genes present in the risk model. The change trajectory of each independent variable was analyzed to determine the optimal model based on the lambda parameter; the figure (Figure 7A) indicates that with the gradual increase of lambda, the number of independent variable coefficients tending to 0 also increases gradually. We used a 10-fold cross-validation technique to build the model and analyzed the confidence interval for each lambda parameter, as shown in Figure 7B. The optimal lambda value was found to be 0.04711966, and 16 genes were selected as target genes based on this value. Based on these 16 genes related to prognosis, nine genes (LPCAT1, SLC2A1, NEIL3, SRXN1, TRNP1, SLC7A11, STC2, ZNF239, CBX2, CDCA8, EPO, PON1, PBK, PFN2, ACOT12, ADH4) were identified using Lasso regression. We developed a final 16-gene signature with a Cox proportional hazards regression model with the RiskScore calculated as follows:

RiskScore = −0.205 × LPCAT1 −0.068 × SLC2A1 −0.194 × NEIL3 + 0.508 × SRXN1 + 0.117 × TRNP 1 + 0.29 × SLC7A11 + 0.209 × STC2 + 0.087 × ZNF239 + 0.25 × CBX2 + 0.111CDCA8 + 0.332EPO + 0.141PON1 + 0.377PBK + 0.186PFN2 + −0.175ACOT12 + −0.052 × ADH4.

We used the TCGA dataset as our training dataset and calculated the risk score of each sample through 16 gene expression levels to analyze the prognostic prediction efficiency classification at 1, 3 and 5 years. The AUC for each year was 0.7. The Riskscore was standardized by zscore, and samples with Riskscore greater than zero after zscore were divided into a high-risk group and a low-risk group. The KM curve was drawn, and we found a significant difference between them (*p* < 0.05) (Figure 7C, 7D). To validate the accuracy of our model, we verified the ICGC dataset using the same method and found similar results (Figure 7E, 7F).

### RiskScore in different clinicopathological features

**Figure 8:**
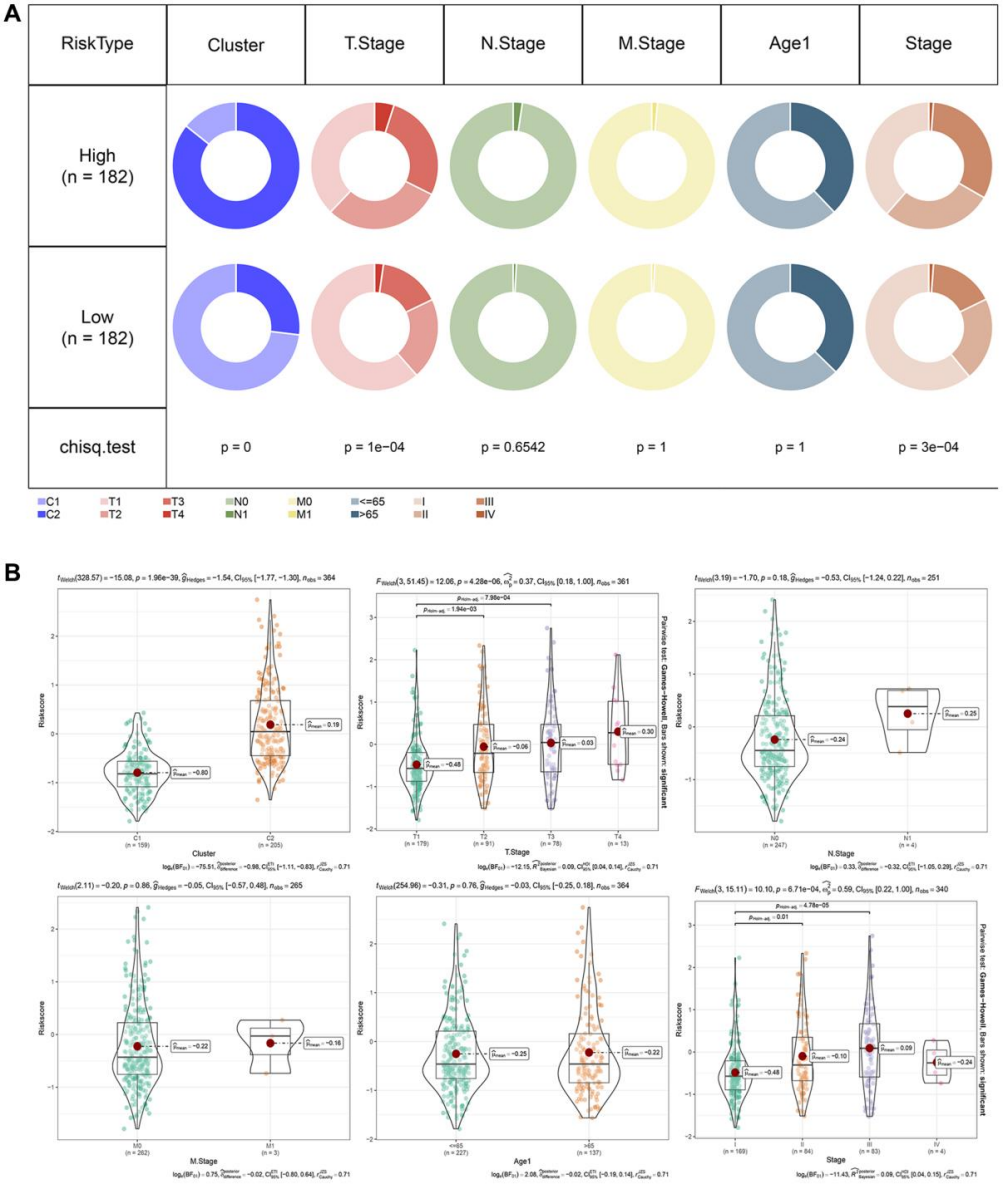
**RiskScore in different clinicopathological features.** (**A**) Comparison of clinical phenotypes between RiskScore groups in the TCGA cohort. (**B**) Differences between RiskScores of different phenotypes in the TCGA cohort (Wilcox. Test, ^*^P < 0.05; ^**^P < 0.01; ^***^P < 0.001; and ^****^P < 0.0001).

We analyzed the relationship between RiskScore scores and tumor clinical characteristics by studying the differences in scores between various clinical phenotypes in the TCGA dataset (Figure 8A). The findings revealed that the risk score increased with clinicopathological characteristics such as clinical grade, as indicated in Figure 8A, 8B.

### Relationship between RiskScore and Immunity

**Figure 9:**
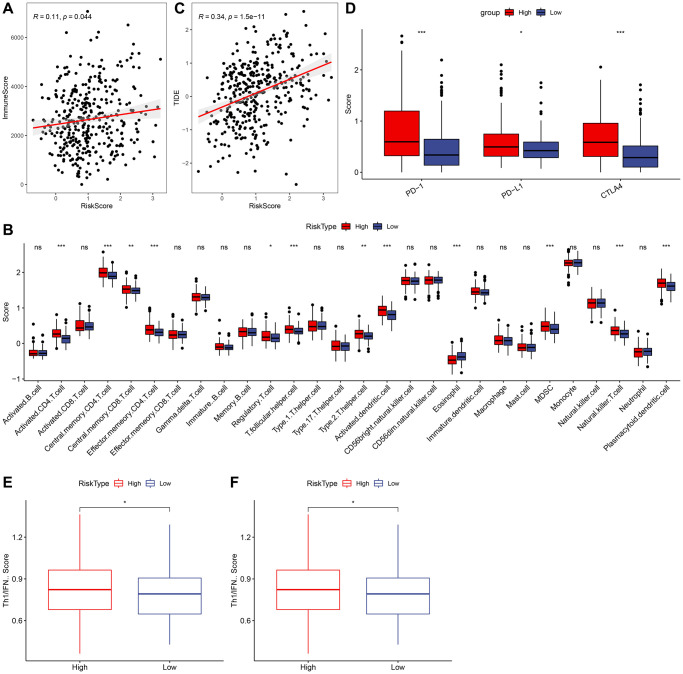
**Relationship between RiskScore and immunity.** (**A**) Correlation analysis of RiskScore and immune score. (**B**) Comparison of 28 immune cell scores in high and low risk groups. (**C**) Correlation analysis of RiskScore for TIDE. (**D**) Comparison of immune checkpoint expression in high and low risk groups. (**E**) Comparison of tumor reactivity scores in high and low risk groups. (**F**) Th1/IFNγ score comparison between high and low risk group.

To further investigate the relationship between RiskScore and immunity, we used ESTIMATE to predict the immunity score and, employing Spearman’s method, found a significant positive correlation between the immunity score and RiskScore, as depicted in Figure 9A. Then we analyzed the scores of 28 immune cells to determine their association with high and low risk groups, discerning significant differences in the scores of 12 immune cells, as shown in Figure 9B. The results of TIDE prediction score demonstrated that the TIDE score was higher among those with higher risk scores, as indicated in Figure 9C. We also evaluated the expression of checkpoints CTLA4, PD-1, and PD-L1 in the high-risk and low-risk groups, finding that the expression of these immune checkpoint genes was higher in the high-risk group (Wilcox. Test, Figure 9D). We calculated the TRS score using the ssGSEA method based on TRS-associated characteristic genes, discovering that the Risk-High group had a higher TRS score (Wilcox. Test, Figure 9E). Furthermore, we computed the interferon (IFN) score using ssGSEA method and identified the gamma score (Th1/IFNγ score) to be significantly higher in the Risk-high group than in the Risk-low group, as presented in Figure 9F.

### RiskScore combined with clinicopathological features further improves the prognostic model and survival prediction

**Figure 10:**
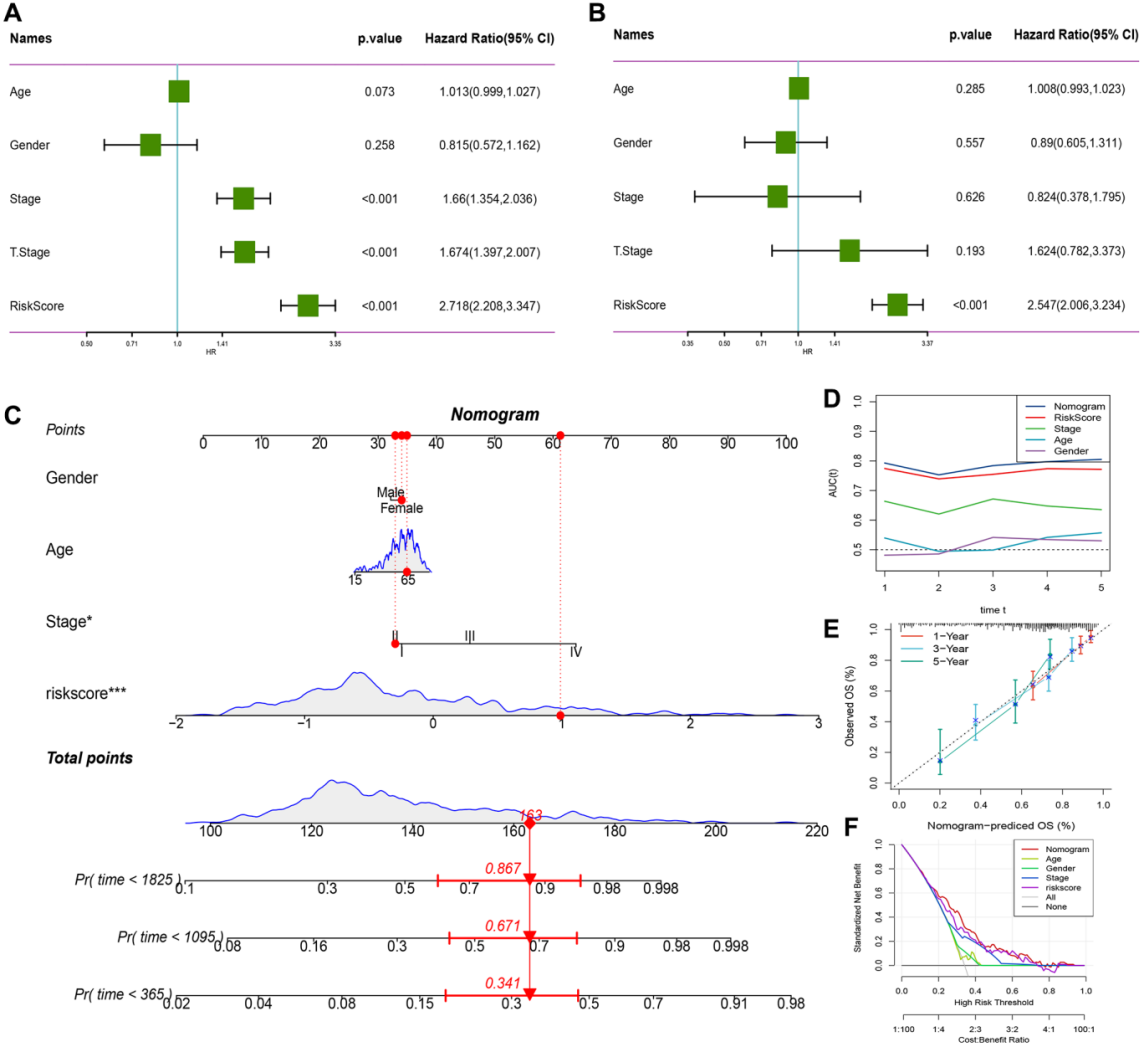
**RiskScore combined with clinicopathological features further improves the prognostic model and survival prediction.** (**A**) Univariate Cox analysis of RiskScore and clinical characteristics. (**B**) Multivariate Cox analysis of RiskScore and clinical characteristics. (**C**) Nomogram model. (**D**) Calibration curve of nomogram at 1, 3 and 5 years. (**E**) Decision curve of nomogram. (**F**) AUC line chart, the horizontal axis is the time unit year, and the vertical axis is the model AUC value.

We demonstrated RiskScore as the most significant prognostic factor by performing univariate and multivariate Cox regression analysis on RiskScore and clinical characteristics, as shown in Figure 10A, 10B. To estimate the risk assessment and survival probability of patients, we integrated RiskScore with other clinicopathological characteristics to design a nomogram presented in Figure 10C. The results of the model showed that RiskScore had the most substantial impact on survival prediction. Moreover, we employed the Decision curve analysis (DCA) to evaluate the model’s reliability and observed that both RiskScore and Nomogram benefits were significantly higher than the extreme curve compared to other clinicopathological features. Both the nomogram and RiskScore demonstrated significant survival predictive power, as presented in Figure 10D, 10F. Additionally, we assessed the prediction accuracy of the model using the Calibration curve, illustrated in Figure 10E, and observed that the calibration curve of the three calibration points at 1, 3, and 5 years was close to the standard curve. This indicates that the nomogram provided good prediction performance.

### Validation of the risk score in HCC

**Figure 11:**
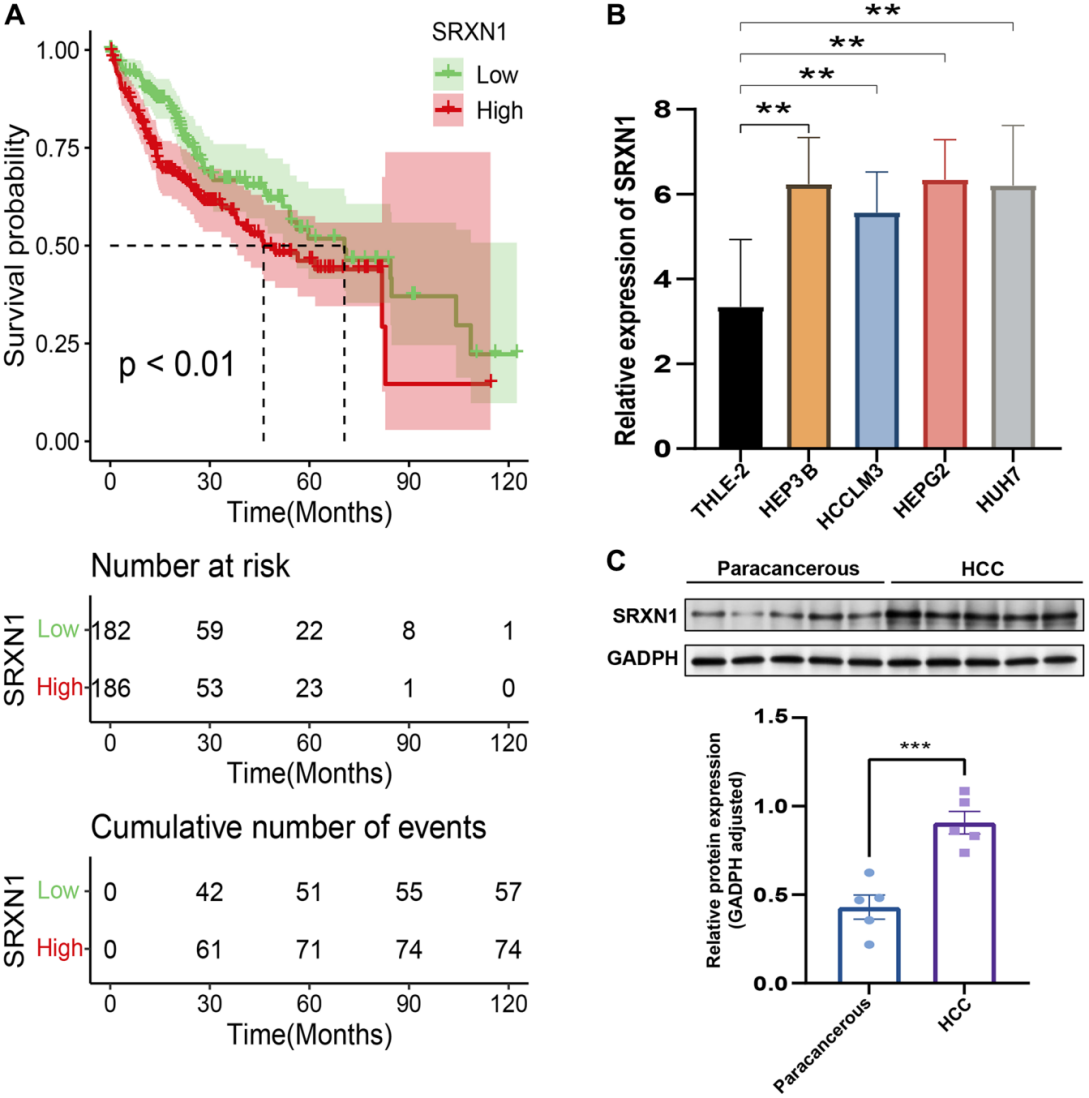
**Validation of the risk score in HCC.** (**A**) Survival analysis of TCGA-LIHC based on SRXN1 levels. (**B**) qRT-PCR images of tumor cell line (HEP3B, HCCLM3, HEPG2 and HUH7). SRXN1 levels were analyzed by qRT-PCR; ^**^P < 0.01 versus liver cell line. (**C**) Representative images of Western blots of the liver cell line (THLE-2) and tumor cell line (HEP3B). SRXN1 levels were determined by Western blot (mean ± SEM; *n* = 5; ^**^P < 0.001 versus THLE-2).

To validate the risk-scoring model in HCC, we first conducted a survival analysis to identify SRXN1 with prognostic significance in the TCGA cohort (Figure 11A). Next, we examined one of the previously identified SRXN1 mRNA that constructs predictive model in several tumor cells. The result of the Quantitative reverse transcriptase-polymerase chain reaction (qRT-PCR) reveals SRXN1 mRNA levels were increased significantly in all tumor cell lines (Figure 11B). Finally, we examined the protein level of SRXN1 in cells of THLE-2 and HEP3B. Our Western blot results show that the SRXN1 protein level was increased significantly in the tumor cell line (Figure 11C).

## DISCUSSION

In our study, we utilized single-cell genotyping to examine ligand-receptor interactions and identify the key receptor-ligand genes in HCC. Based on these genes, we clustered the patients in two clusters with disguising TME characteristics. We then constructed a prognostic model which can creativity predict the prognosis of HCC with 16 genes (receptor-ligand genes). This approach allowed us to gain insight into the patterns of receptor-ligand genotyping within the tumor microenvironment both in intra-tumor and extra-tumor, which could have significant implications for HCC who can be benefits from the immunotherapy.

Ligand-receptor interactions, playing a crucial role for the signal transduction between cancer cells and nonmalignant cells in the tumor microenvironment (TME), is the key to the progression of tumors and exhibit a high degree of heterogeneity, posing challenges in understanding tumor progression and treatment. This heterogeneity is manifested in the interactions within the tumor microenvironment, including the ligand-receptor signaling between tumor cells and non-malignant cells. To resolve it, the clustering method was used in the identifying the special subpopulation for HCC by ligand-receptor genes in our study. A previous study also conducted the comprehensive understanding of these interactions, particularly across 20 solid tumor types, can aid in better understanding the paracrine interactions of cancer cells and the tumor microenvironment [11]. According to previous studies, the intratumoral factors such as hypoxia [12] and metabolic abnormalities [13, 14] may lead to changes in the surface expression of ligands. For instance, in HCC, glycosylation regulator subtypes have been found to be associated with intratumoral factors [15, 16]. In addition, the evolution of hepatocellular carcinoma (HCC) is believed to be driven by both endogenous and exogenous factors, which may include genetic instability (endogenous factors) as well as factors from the tumor microenvironment, such as immune cells and cancer-associated fibroblasts (exogenous factors). Also, some receptors-ligand genes, such as PD-L1/PD1 both expressed in stromal cells or macrophage cells in HCC, could induce the immunosuppressive effect to TME [17, 18]. The aberrant activation of the Wnt signaling pathway, which is initiated by the binding of Wnt ligands to Frizzled receptors, is frequently implicated in stem cell cancer initiation and progression [19].

In our model, we next screened 16 ligand receptor genes in single cell tumor cells through prognosis, and extensive research has officially confirmed the role of these genes in hepatocellular carcinoma. For example, the increased expression of SRXN1 (Sulfiredoxin 1) may be helpful in resisting oxidative stress produced by tumor cells [20]. Overexpression of PBK (PDZ Binding Kinase) may lead to increased proliferation and survival ability of tumor cells [21]. EPO (Erythropoietin) can enhance the survival ability of tumor cells by inhibiting apoptosis and promoting angiogenesis. This may increase the risk of hepatocellular carcinoma [22]. SLC7A11 is involved in the uptake of sulfated amino acids by cells with increasing expression led to increased resistance to chemotherapy drugs in cancer [23].

However, our study is not without limitations. Although the clustering method was useful in identifying specific subpopulations in HCC, further research is needed to validate the clinical relevance of these clusters. In addition, the role of these 16 genes in HCC requires experimental validation to further establish their functional significance in the progression of HCC. Moreover, our prognostic model needs to be validated in larger, prospective studies and across different populations.

In conclusion, our study has shed light on the intricate landscape of ligand-receptor interactions within the TME of HCC. By identifying key receptor-ligand genes and developing a prognostic model, we provide new insights into HCC progression and potential therapeutic targets. Despite the limitations, our findings offer a promising step towards more personalized therapeutic strategies for HCC patients.

## MATERIALS AND METHODS

### Single cell data downloading and preprocessing

We utilized the Seurat (Version 4.1.1) R package to process single cell data obtained from the GEO database (https://www.ncbi.nlm.nih.gov/geo/) for a reliable liver cancer single cell expression profile dataset (GSE149614) [24]. The sequencing platform used was GPL24676 Illumina NovaSeq 6000 for Homo sapiens. We filtered the single-cell data by setting the criteria that each gene should be expressed in at least three cells and each cell should express at least 250 genes. We further ensured that the gene expressed by each cell was more than 500 and less than 4000, the unique molecular identifier (UMI) of each cell was less than 15000, mitochondrial gene content was less than 10%, ribosome expression was more than 3%, and red blood cell expression was less than 1%, using the Percentage Feature Set function. We obtained a total of 18 single-cell sequencing samples from the GSE149614 dataset, including 10 hepatocellular carcinoma tissues and 8 normal liver tissues.

### TCGA database data and ICGC database data download

We retrieved clinical phenotype data of liver cancer from The Cancer Genome Atlas (TCGA) database and removed samples without survival time and survival status. We ensured that all patients had a survival time of more than 0 days. Additionally, we downloaded TCGA expression profile data and obtained 368 tumor samples. Moreover, we downloaded the liver cancer data, expression profile data, and survival data of the ICGC-JP cohort from the International Cancer Genome Consortium (ICGC) database. We removed samples lacking survival time and survival status and ensured that all patients had a survival time of more than 0 days. Eventually, we obtained a total of 232 samples. We calculated snv mutation information of the gene of TCGA-LIHC using Mutect2 software through TCGAbiolinks (version 2.24.3) download [25]. Homologous Recombination Defects, Fraction Altered, Number of Segments, Nonsilent Mutation Rate, Aneuploidy Score, Silent Mutation Rate Data were obtained from a previous study [26].

### Single-cell dimensionality reduction and annotation

To normalize the data of the 18 samples, we applied log-normalization separately. Then, we found hypervariable genes using the FindVariable Features function, which identified variable features based on variance stabilization transformation (VST). We scaled all genes using the ScaleData function followed by PCA dimensionality reduction using RunPCA to find anchor points, choosing dim = 30. The cells were clustered through the FindNeighbors and FindClusters functions with a resolution of 0.45 to avoid batch effect of the samples, a Harmony package (version 0.1.0) was used to batch correct the samples. For cell type annotation, we used the HumanPrimary CellAtlasData dataset in the SingleR (version 1.10.0) R package. We also used the tools Copycat [27] (version 1.0.8). Subsequently, marker genes of each sub-population were screened using the FindAllMarkers function with logfc = 0.5 (fold difference) and Minpct = 0.5 (minimum expression ratio of differential genes), with a screening threshold of a corrected *p*-value less than 0.05.

### Cell communication analysis

We used the R package CellChat (version 1.5.0) for the analysis of cell-to-cell communication [28]. The results of CellChat were used to screen out the receptor ligand genes associated with malignant cells.

### Consistent clustering for receptor-ligand genes

We performed univariate Cox regression analysis to identify genes associated with prognosis (*p* < 0.05), from the genes identified in CellChat. Based on the expression profile of these prognostically relevant genes, we classified the patients using the consensus clustering of tumor tissues in the TCGA dataset through ConsensusClusterPlus (version 1.60.0) package in R programming language [29]. We used the HC algorithm with “Pearson” as the metric distance and performed 500 bootstraps with each bootstrap procedure including 80% of the training set of patients. The number of clusters was set from 2 to 10, and the optimal number of clusters was determined by calculating the consistency matrix and the consistency cumulative distribution function. The CDF was used to determine the best classification.

### Path analysis and variance analysis

We obtained Kyoto Encyclopedia of Genes and Genomes (KEGG) pathway genes from the msigdb (http://www.gsea-msigdb.org/) database. To calculate pathway scores for samples of different molecular subtypes, we utilized the GSVA package [30]. For differential analysis of the pathway scores between the two subtypes, we used the limma package (version 3.52.2) [31]. Here, we selected pathway visualization with |logFC| >0.15 and *P*-value < 0.05 criteria. Furthermore, we conducted differential analysis of gene expression by subtype using the limma package and identified differential genes by screening for |logFC| >log2 (1.2) and *P*-value < 0.05. We then employed gene set enrichment analysis (GSEA) using the Hallmark gene sets in the msigdb database as the background set, and we merged these results with the previous differential analysis to reveal further insights into the pathways and genes involved.

### LASSO regression analysis and risk model construction

Genes significantly associated with survival outcomes were identified through univariate Cox regression models to construct a prognostic model. In addition, reliable predictors were selected using LASSO analysis [32], a compression estimator that constructs a penalty function to compress coefficients and set others to zero. This methodology retains the advantages of subset shrinkage and acts as a biased estimator that reduces multicollinearity problems encountered in regression analysis. We conducted LASSO Cox regression using the R software package glmnet (version 4.1-4). Risk scores for each patient in the TCGA and ICGC databases were calculated using the formula: Risk score = Σ coefficient mRNA × expression level mRNA. Finally, we analyzed the correlation between patient risk scores and prognosis.

### Immune infiltration and prediction of immunotherapy

To assess the distribution of specific cellular components in the immune microenvironment, we calculated the scores of 28 immune cells using ssGSEA based on characteristic genes obtained in a previous study [33]. Differences among subtypes were assessed using Kruskal test. In addition, we assessed immune cell infiltration using ESTIMATE [34] (version 1.0.13). We used TIDE to evaluate the potential clinical effects of immunotherapy across groups [35]. A previous study [36] showed that the TRS score could predict a patient’s response to immunotherapy. CD8+ T cells in the tumor microenvironment can produce interferon-γ (IFNγ), which can up-regulate PD-1/PD-L1 and IDO1 gene expression [37, 38]. Up-regulated IDO1 expression is positively associated with tumor progression and poor prognosis [39]. We extracted Th1/IFNγ gene signatures from a previous study [32] and calculated IFNγ scores using ssGSEA for each patient.

### Cell culture

Here, we purchased normal liver cell line (THLE-2, Shanghai Academy of Life Science), and tumor cell lines, including HEP3B (catalog No. ZQ0024), HEPG2 (catalog No. ZQ0022), HCCLM3 (catalog No. ZQ0023) and HUH7 (catalog No. ZQ0025). Culture dishes with Dulbecco’s Modified Eagle’s Medium (DMEM) containing 10% fetal bovine serum (FBS) were used to inoculate all cell lines to maintain growth at 37°C with5% CO_2_. The cell culture medium was also supplemented with 1% penicillin/streptomycin. The culture dishes were purchased from Guangzhou Jet Biofiltration (Guangzhou, China). FBS, and penicillin/streptomycin were purchased from BI (BI, Ridgefield, CT, USA).

### RNA extraction and quantitative reverse transcription PCR

Using the total RNA isolation kit (Foregene, Chengdu, China) and the PrimeScript RT kit (Takara Biomedical Technology, Beijing, China), RNA was extracted from the cells and reverse transcribed into cDNA. TB Green Premix Ex Taq II (Takara Biomedical Technology, Beijing, China) and was tested by real-time PCR with 2^−ΔΔCt^. GAPDH was the reference gene in the process. The qPCR primers are listed as follows: SRXN1 Forward Primer CAGGGAGGTGACTACTTCTACTC; SRXN1 Reverse Primer CAGGTACACCCTTAGGTCTGA.

### Western blot

RIPA lysis buffer was used to extract the protein, and the BCA Protein Assay Kit was used to calculate the protein concentration. 10% SDS-PAGE was used to separate equal amounts of protein, and the resultant membrane, polyvinylidene difluoride, was used. The membrane was incubated with the primary antibody overnight at 4°C after being blocked with 5% BSA in PBST for 1 hour at room temperature. It was then subjected to three PBST washes before being exposed to an appropriate horseradish peroxidase (HRP)-conjugated secondary antibody for one hour at room temperature. With the use of an ECL chemiluminescent reagent, the reaction was observed. Using Image Lab, the blot’s intensity was measured.

## Supplementary Materials

Supplementary Figure 1

Supplementary Table 1
