# Supplementary material for: Malignant cell receptor-ligand subtypes guide the prediction of prognosis and personalized immunotherapy of liver cancer
Source: Aging (Albany NY). 2024 Jan 18;16(2):1712–32. doi: 10.18632/aging.205453 (PMC10866410; doi:10.18632/aging.205453)
Supplement: Supplementary Figure 1 [file aging-16-205453-s001.pdf]

SUPPLEMENTARY FIGURE

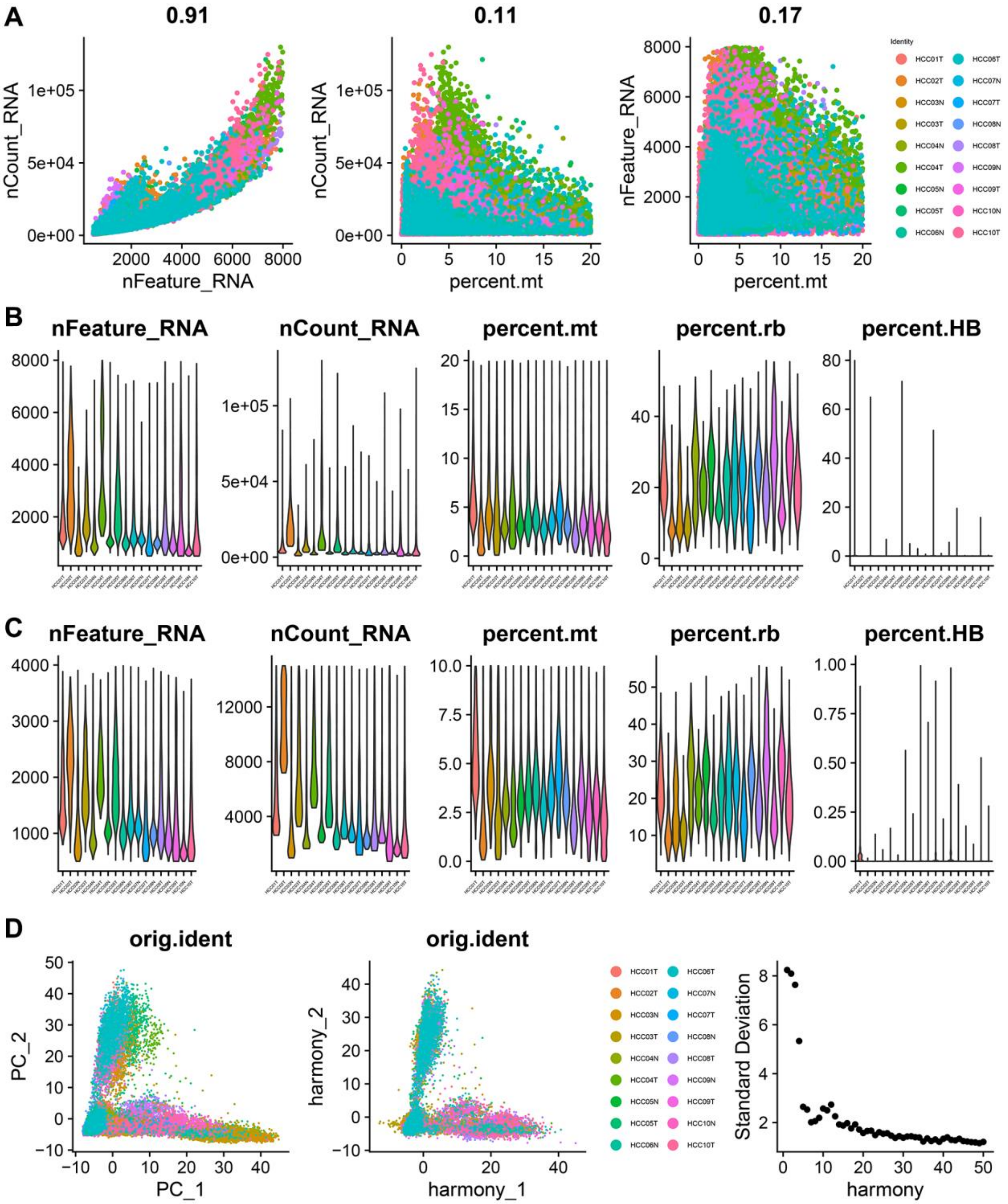

Supplementary Figure 1. (**A**) Relationship between mitochondrial gene and UMI/mRNA quantity, relationship between UMI and mRNA quantity. (**B**) Relationship between mRNA/UMI/mitochondrial content/rRNA content of each sample before filtration. (**C**) Relationship between mRNA/UMI/mitochondrial content/rRNA content of each sample after filtration. (**D**) Sample distribution map of PCA dimension reduction and anchor map of PCA.
