# Supplementary material for: Malignant cell receptor-ligand subtypes guide the prediction of prognosis and personalized immunotherapy of liver cancer
Source: Aging (Albany NY). 2024 Jan 18;16(2):1712–32. doi: 10.18632/aging.205453 (PMC10866410; doi:10.18632/aging.205453)
Supplement: Supplementary Table 1 [file aging-16-205453-s002.pdf]

SUPPLEMENTARY TABLE

Supplementary Table 1. Raw data for western blots and qRT-PCR.

WB			
	**GADPH**	**SRXN1**	**GAPDH/SRXN1**
1	99187.844	35326.844	0.356161023
2	84853.137	18479.309	0.217779915
3	88187.844	41430.673	0.469800271
4	94555.966	59051.551	0.62451428
5	95978.966	46234.602	0.481715983
6	96021.43	104183.673	1.085004389
7	107641.258	79371.137	0.737367237
8	101272.258	87097.844	0.860036556
9	86776.894	72204.016	0.832064996
10	85029.53	86875.187	1.021706071

qPCR				
**THLE-2**	**HEP3B**	**HCCLM3**	**HEPG2**	**HUH7**
3.082	5.122	5.103917211	7.839581737	5.285772093
4.053	7.378	7.032982417	6.364222221	4.958378712
2.902	7.542	5.468909092	6.374691752	5.459431619
6.146	5.861	5.56924803	5.185866545	8.572624443
2.054	6.522	6.063934306	6.763145958	7.275472718
1.806	4.987	4.164303583	5.502712486	5.656782364
